# Why Make Things Complicated When They Can Be Simple? Case Series and Systematic Review on the Reconstruction of Full-Thickness Soft-Tissue Heel Defects

**DOI:** 10.3390/jcm15134899

**Published:** 2026-06-24

**Authors:** Aurélie Cavin, Julie Triolo, Yves Harder, Jérémy Brühlmann

**Affiliations:** 1 Department of Plastic, Reconstructive and Aesthetic Surgery and Hand Surgery, Centre Hospitalier Universitaire Vaudois (CHUV), 1011 Lausanne, Switzerland; julie.triolo90@gmail.com (J.T.); yves.harder@chuv.ch (Y.H.); jeremy.bruhlmann@chuv.ch (J.B.); 2 Faculty of Biology and Medicine, University of Lausanne, 1015 Lausanne, Switzerland

**Keywords:** case series, heel defect, heel defect local coverage, Limberg flap, local flaps, reconstructive surgery, rhomboid flap, soft-tissue defect

## Abstract

**Background/Objectives**: Reconstruction of full-thickness soft-tissue defects of the heel can be challenging due to the specific structural and functional demands of this region. Local flaps are often used due to their ability to provide durable and sensate coverage. This case series and systematic review aim to assess their surgical efficacy and reported outcomes, particularly in the context of the rhomboid flap. **Methods**: A systematic review was conducted in accordance with PRISMA guidelines, using PubMed, Cochrane and EBSCO. Studies published up to March 2026 evaluating local flaps were included, whereas distant pedicled and microvascular flaps were excluded. Defect size, flap types and surgical outcome were extracted and synthesized in a comparative table. In addition, we present four clinical cases of full-thickness soft-tissue heel defects reconstructed with a local rhomboid flap. This retrospective, single-center case series includes patients treated at our institution between January 2023 and March 2026, with initial debridement followed by flap coverage. **Results**: The four patients had a mean defect size of 4.1 cm^2^. All defects ultimately healed, though one case demonstrated delayed wound healing. Eventually, neither donor-site morbidity, nor recurrence were observed during a mean follow-up of 7.4 months (range 1 to 17 months). Nine studies were included in the review, encompassing 56 patients. Despite the variety of the studies regarding design and flaps used, all focused on outcomes, including flap survival, complication rate, and functional recovery. Local flaps appear to be a feasible option for this type of soft-tissue defect; however, they seem to be limited to small defects. **Conclusions**: Local flaps may represent a valuable option for small full-thickness heel defects up to 6 cm^2^ according to the “like-with-like” principle. They are associated with low surgical morbidity and do not compromise subsequent reconstructive options, making them a reasonable first-line approach. Limitations include the small sample size, retrospective design, the unequal follow-up time, as well as the absence of standardized functional outcome assessment.

## 1. Introduction

**Figure 1:**
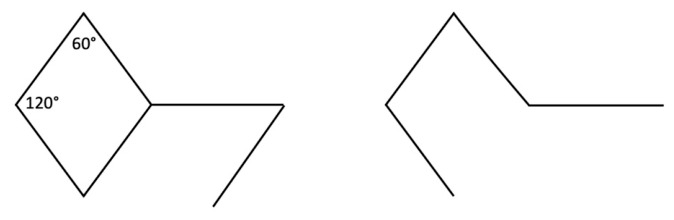
Schematic illustration of a rhomboid (Limberg) flap.

The reconstruction of soft-tissue heel defects remains challenging due to the unique structural and functional demands of this region, where options such as advancement flaps are limited by the tight and inelastic nature of the plantar glabrous skin [1]. While healing by secondary intention may be an option, it is often time-consuming, requires frequent dressing changes and can result in unstable scarring [2]. In this context, small local flaps such as rhomboid flaps may represent a practical solution. Local flaps provide durable coverage with tissue that matches the structural properties (“like-with-like”) and therefore the functional and mechanical properties of the area to be reconstructed. The main advantages of the rhomboid flap include its versatility and technical simplicity. It can be used to reconstruct defects of different sizes and locations. The flap is usually based on four equal sides in length with two acute angles of 60° and two obtuse angles of 120°, which allows for a transposition of the skin into the defect [3] (Figure 1).

Originally described by Alexander Limberg, this flap is designed as a rhombus-shaped transposition flap, making it particularly effective for closing defects with minimal tension, while preserving local tissue characteristics [4]. The flap has a random-pattern vascular supply derived from the dermal and subdermal plexuses [5]. This vascular network is located between the hypodermis and the reticular dermis, and preservation of adequate subcutaneous tissue during flap elevation may significantly enhance flap viability [6]. Kang et al. also reported excellent long-term scar integration and high patient satisfaction following rhomboid flap reconstruction, with no major postoperative complications observed [7]. Despite these advantages, rhomboid flaps may be less suitable in areas with limited skin mobility or increased mechanical tension, where excessive closure tension or suboptimal flap design may impair wound healing and negatively affect scar quality or flap viability [6]. Various modifications have been introduced to optimize tension distribution and aesthetic outcome, especially in challenging anatomical areas [8].

In this context, the aim of this study was to analyze the use of a rhomboid flap as a simple solution for addressing small and well-circumscribed full-thickness soft-tissue defects of the heel based on a personal case series.

In addition, a systematic review of the current literature was undertaken to evaluate the effectiveness and the surgical outcome of this flap on this specific surgical indication.

## 2. Materials and Methods

### 2.1. Literature Search

A review protocol was developed based on the preferred reporting items for systematic reviews and meta-analyses (PRISMA) statement. A comprehensive literature search was performed in the literature databases PubMed, Cochrane Library and EBSCO until March 2026, to include all relevant studies. Articles in all languages were considered (English, German, Chinese) (Supplementary Materials).

The following research terms were used: “local flap AND heel defect” or “Limberg AND heel defect” or “rhomboid AND heel defect” and “local coverage AND heel defect”. Randomized controlled trials, case–control studies, prospective and retrospective cohort studies, as well as case series were included.

**Figure 2:**
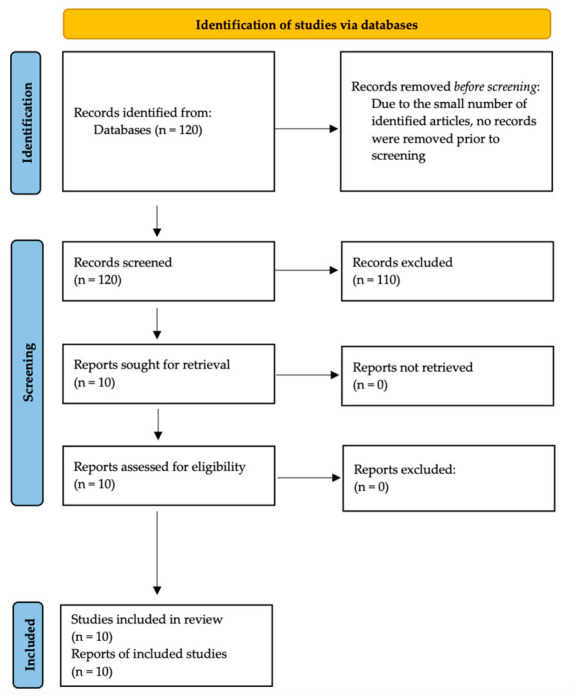
PRISMA flow diagram illustrating the study selection process.

Studies that did not focus on options using local flaps for the defect reconstruction, including distant pedicled flaps or microvascular flaps, or studies evaluating defects beyond the area of the heel were excluded (Figure 2).

Although no formal risk-of-bias tool was applied, potential bias in the included studies was minimized by having two reviewers screen and evaluate each study (A.C. and J.B.).

The protocol for this case series was approved by the CER-VD Ethics Committee (registration number 2025-00277) and the review was registered in PROSPERO (International Prospective Register of Systematic Reviews; CRD420251266790).

### 2.2. Data Extraction

Two independent reviewers screened titles and abstracts, followed by full-text assessment of eligible studies. Articles meeting exclusion criteria were removed. Relevant studies were recorded using Zotero (version 7.0.11, 2024, George Mason University, Fairfax, VA, USA) and summarized in Table 1. Extracted data included the number of patients, defect size, type of local flap, and reported outcomes such as flap survival and complications.

Defect size was reported as mean values from individual studies without further statistical analysis. Missing or unclear data were considered not reported unless otherwise specified. Due to the small number of studies and heterogeneity in flap types and defect sizes, no meta-analysis was performed, and results were synthesized narratively.

### 2.3. Case Series

The retrospective single-center case series included patients with small, well-circumscribed full-thickness soft-tissue heel defects suitable for local flap coverage. Patients requiring distant pedicled or free flap reconstruction were excluded.

All patients were identified from the surgical records of the Department of Plastic, Reconstructive and Aesthetic Surgery at the Centre Hospitalier Universitaire Vaudois (CHUV), Lausanne, Switzerland, and were treated between January 2023 and March 2026. A two-stage approach was indicated for two of them, including first surgical debridement followed by temporary coverage with a negative pressure wound therapy (NPWT) device. The procedures were performed under general anesthesia with the patient in a prone position by a board-certified plastic surgeon. Thereafter, defect closure was performed using a rhomboid-shaped local flap elevated in a pre-fascial plane, followed by tension-free layered closure with absorbable sutures for the subcutaneous tissue and non-absorbable sutures for the skin. The terms “rhomboid-shaped” and “rhomboid-like” were preferred in this manuscript, as flap design followed the principles of a classical rhomboid flap (or Limberg flap) without strict geometric angle measurement. In two cases, the donor site required split-thickness skin grafting. Standard dressings were applied, and sutures were maintained for at least 3 weeks. All cases were managed according to the department’s standard operative practice for heel reconstruction. This case series has been reported in line with the PROCESS guidelines (Preferred Reporting Of Case Series in Surgery) [18].

## 3. Results

### 3.1. Case Series

#### 3.1.1. Case 1

A 63-year-old patient presented with abscessed lesions on the plantar surface of the forefoot and the heel after stepping on broken glass. In this context an initial presentation of diabetes mellitus was diagnosed.

After surgical debridement and application of NPWT, the medial plantar wound was covered with a split-thickness skin graft, while the heel defect (1.5 × 1.5 cm) was reconstructed using a local rhomboid-like flap.

**Figure 3:**
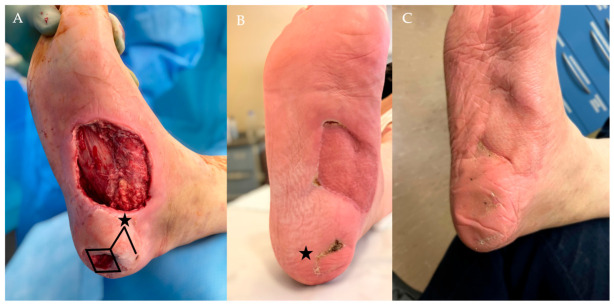
Case 1. (**A**) Intraoperative view after surgical debridement of a 1.5 × 1.5 cm defect with the planning of the foreseen local rhomboid-like flap. The asterix indicates the corresponding flap corner before (**A**) and after (**B**) flap transposition at 5 weeks follow-up. (**C**) 3 months follow-up.

The postoperative course was uneventful, showing stable soft tissues at 5 weeks and 3 months after surgery. Follow-up at 4.8 months showed stable flap and scar (Figure 3).

#### 3.1.2. Case 2

**Figure 4:**
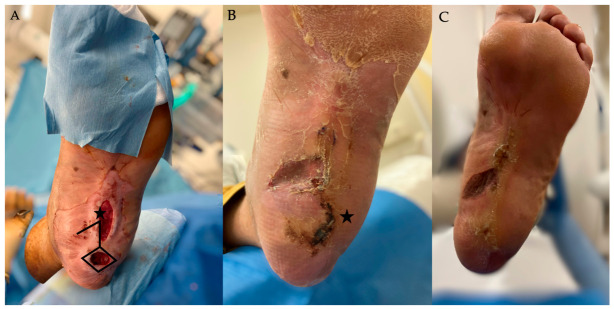
Case 2. (**A**) Intraoperative view after surgical debridement of a 2 × 1 cm defect with the planning of the foreseen local rhomboid-like flap. The asterix indicates the corresponding flap corner before (**A**) and after (**B**) flap transposition at 4 weeks follow-up. (**C**) 12 months follow-up.

A 59-year-old civil engineer, previously treated for osteitis of the calcaneus, presented with an abscess caused by trauma on a pre-existing foot deformity. Multiple surgical drainages and debridements resulted in a full-thickness soft-tissue defect on the plantar surface and the heel (2 × 1 cm). Laxity of the surrounding tissues was limited due to pre-existing scars resulting from prior debridements and secondary healing of the wounds. Accordingly, defect coverage was performed using a local rhomboid-like flap and split-thickness skin graft closure of the adjacent donor site. The postoperative course was uneventful, showing stable soft tissues at 4 weeks and 12 months (Figure 4).

#### 3.1.3. Case 3

A 78-year-old patient, with multiple comorbidities and left-sided hemiparesis following a stroke, developed a stage III pressure sore according to the National Pressure Injury Advisory Panel (NPIAP) on the posterior, non-weight-bearing area of the left heel. After surgical debridement, defect reconstruction (2 × 3 cm) was performed using a local rhomboid-like flap and primary closure of the donor site. The postoperative course was uneventful, showing good wound healing at 1.3 months. The patient was subsequently lost to follow-up due to deterioration of her general condition.

#### 3.1.4. Case 4

A 66-year-old patient, active smoker, presented with an unstable scar over a pressure ulcer healed by secondary intention that developed after post-traumatic immobilization. The scar showed recurrent ulceration since 2022. Osteomyelitis was excluded. After surgical debridement, defect reconstruction (2 × 3 cm) was performed using a local rhomboid-like flap and split-thickness skin grafting of the donor site. Delayed wound healing occurred postoperatively, but healing was achieved after 4 months and the patient no longer required dressings.

An overview of all cases is provided in Table 2.

### 3.2. Literature Search

The literature search identified 118 results in PubMed, 1 in Cochrane, and 1 in EBSCO, with no additional relevant results using more specific terms such as Limberg AND heel defect or rhomboid AND heel defect. Of these, nine articles met the inclusion criteria, comprising a total of 56 patients, while 111 articles were excluded as they did not evaluate local flaps. Among the included studies, the overall reported complication rate was approximately 9%. An additional search using the terms “heel defect AND local coverage” yielded 50 PubMed results, of which 5 met the criteria and had already been identified (Figure 2). Details of included studies are summarized in Table 1.

Huang et al. described a modified V-Y advancement flap to cover only the posterior area of the distal third of the lower leg, including the Achilles tendon insertion. Their technique proved promising for wounds ranging from 2 × 2 cm to 5 × 10 cm. One flap developed necrosis, whereas all other flaps (*n* = 21) showed uneventful healing with preserved sensation [9].

Several authors have proposed decision trees or algorithms to guide the reconstruction of defects of the heel. Eisenschenk et al. developed an algorithm for the reconstruction of defects of the distal lower leg in general, as well as the ankle and the heel in particular. Thereby, local flaps were considered an option only for soft-tissue defects not exceeding a size of approximately 4 × 4 cm and 11 cases were reported. At this point, it has to be said that the instep flap was not considered because it is usually used as an island flap, and therefore an exclusion criterion. Among these, four soft-tissue defects were located at the posterior area of the heel and were treated using two transpositional flaps and two rotational flaps. No complications were reported [10]. Similarly, Krishna et al. proposed a decision tree using V-Y advancement or rotation flaps for small anterior weight-bearing defects up to 3 cm, and advancement of avulsed skin for posterior non-weight-bearing defects (mean size ~7 × 5 cm). One of six patients developed partial necrosis, which healed after debridement and secondary closure [11]. El-Shazly et al. incorporated local flaps into their reconstructive algorithm for heel defects smaller than 3 cm^2^, acknowledging their suitability when local perfusion is adequate; however, these techniques were not specifically represented as a distinct group within their reported clinical cohort [12]. Menke et al. published two studies proposing a decision tree where soft-tissue defects of the heel measuring between 3 and 5 cm in diameter could be treated using local rotation flaps. The authors underlined the fact that the placement of the incision to harvest the flap within the weight-bearing area had to be considered as a disadvantage. Nevertheless, Menke et al. emphasized the benefit of maintaining sensitivity by transposing glabrous skin into the defect, matching structure and function of the needed skin. In their second study, six patients were treated with local flaps, and the overall complication rate was 17% (1/6) with one case of total flap loss. Complications were more frequent in the loco-regional flap group compared to the free flap group [13,14].

Yetkin et al. used bilobed transposition flaps in 12 patients with small round defects (mean diameter 1.6 cm), including 4 on the heel. Eleven healed uneventfully within 3 weeks, while one required surgical revision [15].

Singh et al. treated a total of 16 patients with full-thickness skin defects all located at the weight-bearing area of the heel. Three defects were covered with a local V-Y advancement flap. They reported excellent results. In one case, surgical delay was required before final closure by flap advancement, with a two-stage procedure consisting of initial flap elevation followed by advancement a few days later to improve flap survival through hypoxia-induced arteriogenesis and angiogenesis [16,19].

Finally, Ding et al. reported rotation flaps in nine patients with weight-bearing heel defects ranging from 2 × 4 cm to 6 × 8 cm, with complete flap survival [17].

## 4. Discussion

The findings of the four presented cases suggest that small, well-circumscribed full-thickness soft-tissue heel defects can be effectively reconstructed using local flaps, particularly when defects do not exceed 6 cm^2^. The procedure is relatively simple and less invasive, which may be advantageous as these patients are often elderly and present with multiple comorbidities, making more complex procedures such as regional or microvascular flaps less suitable. The current literature includes 56 patients meeting the criteria for inclusion between 1999 and 2026. This limited number may reflect a tendency to favor either secondary healing or more complex reconstructive options, especially in patients with poor general condition or at risk for wound healing complications [14].

These observations highlight the limited evidence regarding the use of local flaps for heel reconstruction and support the relevance of the present study. Local flaps should therefore be considered among the reconstructive options for selected patients. Despite the weight-bearing nature of the heel, they provide effective coverage for defects that would otherwise require prolonged wound care or more complex procedures, while respecting the “like-with-like” principle and avoiding additional donor-site morbidity [1]. Recent reconstructive algorithms proposed by Crowe et al. and Krishna et al. emphasize that heel reconstruction should be tailored to defect size, location, tissue requirements, and patient factors, with local flaps remaining appropriate options for small and relatively simple defects, while larger or more complex wounds may require regional or free flap reconstruction [11,20]. Rhomboid-like local flaps may therefore represent a simple and functionally appropriate first-line option for selected heel defects as recently reported by Elhaddad et al., who could demonstrate a successful reconstruction of a plantar midfoot ulcer (4 × 4 cm) using a Dufourmentel rhomboid flap, with stable healing in the weight-bearing area and low donor-site morbidity. Accordingly, their experience further supports the use of rhomboid-based local flaps for plantar defects [21].

This study has several limitations. The evidence is based on small and heterogeneous patient cohorts with varying defect etiologies and reconstructive techniques. Our institutional case series was retrospective and included a limited number of patients with follow-up durations ranging from 1 to 17 months. In addition, one patient was lost to follow-up, and neither standardized functional, nor patient-reported outcome measures were used. Finally, the review process involved only two reviewers without formal assessment of bias. Although these limitations restrict the strength and generalizability of the herein-presented conclusions, the preliminary clinical outcomes observed in both the institutional experience and the literature support the fact that local flaps may be suitable for selected heel defects and add further clinical evidence to recent reports such as those published by Elhaddad et al. [21]. Future studies with larger cohorts, longer follow-up time, and standardized functional evaluation are needed to better assess long-term outcomes.

## 5. Conclusions

Local skin flaps may represent a useful option for selected small full-thickness heel defects involving both weight-bearing and non-weight-bearing areas. They provide “like-with-like” reconstruction with minimal donor-site morbidity.

However, their indication is limited by defect size up to approximately 6 cm^2^, as demonstrated in this series, and location of the defect. If used as a first-line option and despite partial healing, local flaps do not compromise subsequent reconstructive options, supporting the consideration of a simple reconstructive option before progressing to more complex procedures, according the reconstructive ladder principle rather than the reconstructive elevator principle. This aspect is particularly important, since many of these patients present with multiple comorbidities [22,23].

The systematic review identified only nine studies over more than two decades. This scarcity of data highlights the need for further clinical reports to better define the indications and limitations of these techniques.

## Figures and Tables

**Table 1:** Included studies.

Authors and Years	Country and Language	Total Number of Patients	Total Number of Heel Defects Treated with Local Skin Flaps	Defect Size (cm)	Type of Flap	Outcomes (%)
Huang et al. (2023) [9]	China, English	22	21	2.1 × 2 to 5 × 10	Modified V-Y advancement	1 tip necrosis (4.7)
Eisenschenk et al. (2006) [10]	Germany, German	81	4	Mean: 3 × 2, not defined in 3 cases	2× transposition, 2× rotation	No complication
Krishna et al. (2021) [11]	India, English	40	6	7 × 5.2	V-Y advancement, rotation, advancement	1 tip necrosis (16)
El-Shazly et al. (2008) [12]	Egypt, English	46	No enrolled patient was treated using local flaps	<3	0	Not known
Menke et al. (2000) [13]	Germany, German	52	3	4 × 5	1× rotation, 1× advancement, 1× transposition	1 complete necrosis (33) (advancement flap)
Menke et al. (2001) [14]	Germany, German	44	6	3 × 6	Transposition	1 total flap loss (17)
Yetkin et al. (2003) [15]	Turkey, English	12	4	1–3.2	Bilobed	1 wound dehiscence (25)
Singh et al. (2018) [16]	India, English	16	3	3 × 3 to 4 × 5	V-Y advancement	Uneventful healing
Ding et al. (1999) [17]	China, Chinese	9	9	2 × 4 to 6 × 8	Rotation	No complication

**Table 2:** Summary of presented cases.

Patient	Occupation	Comorbidities	History	Defect Size (cm × cm)	Time to Complete Healing (Weeks)	Outcomes
1	Retired	Diabetes type 2, hypertension, obesity, malnutrition	Traumatic wound with abscess	1.5 × 1.5	3	No recurrence of wounds at 4.8 months
2	Civil engineer	Hypertension	Traumatic wound with abscess, pre-existing plantar foot deformity following trauma in childhood	2 × 1	4	No recurrence of wounds at 12 months
3	Retired	Hypertension, cardiac failure, malnutrition, hypercholesterolemia	Pressure ulcer after stroke	2 × 3	3	Lost to follow-up after 1.3 months
4	Retired	Active smoker	Pressure ulcer following post-traumatic immobilization	2 × 3	16	No recurrence of wounds at 5.6 months

## Data Availability

Data are not publicly available due to patient privacy and ethical restrictions.

## Supplementary Materials

The following supporting information can be downloaded at: https://www.mdpi.com/article/10.3390/jcm15134899/s1, File S1: PRISMA 2020 checklist.

## Abbreviations

The following abbreviations are used in this manuscript:

PRISMA	Preferred Reporting Items for Systematic Reviews and Meta-Analyses
CER-VD	Ethics Committee of the Canton of Vaud
CHUV	Centre Hospitalier Universitaire Vaudois
NPWT	Negative Pressure Wound Therapy
