# Supplementary material for: Why Make Things Complicated When They Can Be Simple? Case Series and Systematic Review on the Reconstruction of Full-Thickness Soft-Tissue Heel Defects
Source: J Clin Med. 2026 Jun 24;15(13):4899. doi: 10.3390/jcm15134899 (PMC13361478; doi:10.3390/jcm15134899)
Supplement: Supplementary file 1 [file jcm-15-04899-s001.zip › File S1.pdf]

PRISMA

PRISMA 2020 for Abstracts Checklist

Section and Topic	Item #	Checklist item	Reported (Yes/No)
**TITLE**			
Title	1	Identify the report as a systematic review.	Y
**BACKGROUND**			
Objectives	2	Provide an explicit statement of the main objective(s) or question(s) the review addresses.	Y
**METHODS**			
Eligibility criteria	3	Specify the inclusion and exclusion criteria for the review.	Y
Information sources	4	Specify the information sources (e.g. databases, registers) used to identify studies and the date when each was last searched.	Y
Risk of bias	5	Specify the methods used to assess risk of bias in the included studies.	No formal risk-of-bias assessment was performed
Synthesis of results	6	Specify the methods used to present and synthesise results.	Y
**RESULTS**			
Included studies	7	Give the total number of included studies and participants and summarise relevant characteristics of studies.	Y
Synthesis of results	8	Present results for main outcomes, preferably indicating the number of included studies and participants for each. If meta-analysis was done, report the summary estimate and confidence/credible interval. If comparing groups, indicate the direction of the effect (i.e. which group is favoured).	Y
**DISCUSSION**			
Limitations of evidence	9	Provide a brief summary of the limitations of the evidence included in the review (e.g. study risk of bias, inconsistency and imprecision).	Y Conclusion
Interpretation	10	Provide a general interpretation of the results and important implications.	Y Conclusion
**OTHER**			
Funding	11	Specify the primary source of funding for the review.	Funding information is not included in the abstract but at the enf of the manuscript

PRISMA 2020 for Abstracts Checklist

Section and Topic	Item #	Checklist item	Reported (Yes/No)
Registration	12	Provide the register name and registration number.	PROSPERO registration number is not included in the abstract but in the manuscript.

From: Page MJ, McKenzie JE, Bossuyt PM, Boutron I, Hoffmann TC, Mulrow CD, et al. The PRISMA 2020 statement: an updated guideline for reporting systematic reviews. BMJ 2021;372:n71. doi: 10.1136/bmj.n71. This work is licensed under CC BY 4.0. To view a copy of this license, visit https://creativecommons.org/licenses/by/4.0/

PRISMA

PRISMA 2020 Checklist

Section and Topic	Item #	Checklist item	Location where item is reported
**TITLE**			
Title	1	Identify the report as a systematic review.	Y
**ABSTRACT**			
Abstract	2	See the PRISMA 2020 for Abstracts checklist.	Y
**INTRODUCTION**			
Rationale	3	Describe the rationale for the review in the context of existing knowledge.	Y paragraphs 1, 4, 5
Objectives	4	Provide an explicit statement of the objective(s) or question(s) the review addresses.	Y paragraphs 4, 5
**METHODS**			
Eligibility criteria	5	Specify the inclusion and exclusion criteria for the review and how studies were grouped for the syntheses.	Y paragraphs 2, 3
Information sources	6	Specify all databases, registers, websites, organisations, reference lists and other sources searched or consulted to identify studies. Specify the date when each source was last searched or consulted.	Y paragraphs 1, 2
Search strategy	7	Present the full search strategies for all databases, registers and websites, including any filters and limits used.	Y paragraphs 1, 2
Selection process	8	Specify the methods used to decide whether a study met the inclusion criteria of the review, including how many reviewers screened each record and each report retrieved, whether they worked independently, and if applicable, details of automation tools used in the process.	Y *2.2 data extraction*
Data collection process	9	Specify the methods used to collect data from reports, including how many reviewers collected data from each report, whether they worked independently, any processes for obtaining or confirming data from study investigators, and if applicable, details of automation tools used in the process.	Y *2.2 data extraction*
Data items	10a	List and define all outcomes for which data were sought. Specify whether all results that were compatible with each outcome domain in each study were sought (e.g. for all measures, time points, analyses), and if not, the methods used to decide which results to collect.	Y *2.2 data extraction*
	10b	List and define all other variables for which data were sought (e.g. participant and intervention characteristics, funding sources). Describe any assumptions made about any missing or unclear information.	Y *2.2 data extraction* paragraph 2
Study risk of bias assessment	11	Specify the methods used to assess risk of bias in the included studies, including details of the tool(s) used, how many reviewers assessed each study and whether they worked independently, and if applicable, details of automation tools used in the process.	Partial. *2.2 data extraction.* No formal risk-of-bias assessment tool was applied due to the small number and heterogeneity of included studies.
Effect measures	12	Specify for each outcome the effect measure(s) (e.g. risk ratio, mean difference) used in the synthesis or presentation of results.	Partial. *2.2*

PRISMA 2020 Checklist

Section and Topic	Item #	Checklist item	Location where item is reported
			data extraction. Due to the small number of studies and heterogeneity in flap types and defect sizes, no meta-analysis was performed, and results were synthesized narratively.
Synthesis methods	13a	Describe the processes used to decide which studies were eligible for each synthesis (e.g. tabulating the study intervention characteristics and comparing against the planned groups for each synthesis (item #5)).	Y 2.1 & 2.2
	13b	Describe any methods required to prepare the data for presentation or synthesis, such as handling of missing summary statistics, or data conversions.	Y 2.2
	13c	Describe any methods used to tabulate or visually display results of individual studies and syntheses.	Y 2.2 & Table 1
	13d	Describe any methods used to synthesize results and provide a rationale for the choice(s). If meta-analysis was performed, describe the model(s), method(s) to identify the presence and extent of statistical heterogeneity, and software package(s) used.	Y 2.2<br>A narrative synthesis was performed due to the small number and heterogeneity of studies.
	13e	Describe any methods used to explore possible causes of heterogeneity among study results (e.g. subgroup analysis, meta-regression).	N No subgroup analysis was performed.
	13f	Describe any sensitivity analyses conducted to assess robustness of the synthesized results.	N No sensitivity analyses were conducted.
Reporting bias	14	Describe any methods used to assess risk of bias due to missing results in a synthesis (arising from reporting biases).	N No formal

Section and Topic	Item #	Checklist item	Location where item is reported
assessment			assessment of reporting bias was performed.
Certainty assessment	15	Describe any methods used to assess certainty (or confidence) in the body of evidence for an outcome.	N No formal certainty assessment was performed.
**RESULTS**			
Study selection	16a	Describe the results of the search and selection process, from the number of records identified in the search to the number of studies included in the review, ideally using a flow diagram.	Y 3.1 & Figure 2
	16b	Cite studies that might appear to meet the inclusion criteria, but which were excluded, and explain why they were excluded.	Partial. 3.2 Individual excluded studies are not listed.
Study characteristics	17	Cite each included study and present its characteristics.	Y 3.2 & Table 2
Risk of bias in studies	18	Present assessments of risk of bias for each included study.	No formal risk-of-bias assessment was performed
Results of individual studies	19	For all outcomes, present, for each study: (a) summary statistics for each group (where appropriate) and (b) an effect estimate and its precision (e.g. confidence/credible interval), ideally using structured tables or plots.	Y 3.2 & Table 2
Results of syntheses	20a	For each synthesis, briefly summarise the characteristics and risk of bias among contributing studies.	Partial<br>Study heterogeneity and limitations are discussed, but no formal risk-of-bias assessment was performed.
	20b	Present results of all statistical syntheses conducted. If meta-analysis was done, present for each the summary estimate and its precision (e.g. confidence/credible interval) and measures of statistical heterogeneity. If comparing groups, describe the direction of the effect.	Not applicable, No quantitative

PRISMA

PRISMA 2020 Checklist

Section and Topic	Item #	Checklist item	Location where item is reported
			synthesis was conducted.
	20c	Present results of all investigations of possible causes of heterogeneity among study results.	No formal heterogeneity analysis was performed
	20d	Present results of all sensitivity analyses conducted to assess the robustness of the synthesized results.	No sensitivity analyses were conducted.
Reporting biases	21	Present assessments of risk of bias due to missing results (arising from reporting biases) for each synthesis assessed.	No bias assessment was performed.
Certainty of evidence	22	Present assessments of certainty (or confidence) in the body of evidence for each outcome assessed.	No formal certainty assessment was performed.
**DISCUSSION**			
Discussion	23a	Provide a general interpretation of the results in the context of other evidence.	Y paragraphs 1, 2
	23b	Discuss any limitations of the evidence included in the review.	Y paragraph 3
	23c	Discuss any limitations of the review processes used.	Y paragraph 3
	23d	Discuss implications of the results for practice, policy, and future research.	Y paragraph 2 & Conclusions
**OTHER INFORMATION**			
Registration and protocol	24a	Provide registration information for the review, including register name and registration number, or state that the review was not registered.	Y 2.1
	24b	Indicate where the review protocol can be accessed, or state that a protocol was not prepared.	Y 2.1
	24c	Describe and explain any amendments to information provided at registration or in the protocol.	No amendments were made to the registered protocol
Support	25	Describe sources of financial or non-financial support for the review, and the role of the funders or sponsors in the review.	Y *Funding*

PRISMA 2020 Checklist

Section and Topic	Item #	Checklist item	Location where item is reported
Competing interests	26	Declare any competing interests of review authors.	Y *Conflicts of Interest*
Availability of data, code and other materials	27	Report which of the following are publicly available and where they can be found: template data collection forms; data extracted from included studies; data used for all analyses; analytic code; any other materials used in the review.	Y *Data Availability Statement*

From: Page MJ, McKenzie JE, Bossuyt PM, Boutron I, Hoffmann TC, Mulrow CD, et al. The PRISMA 2020 statement: an updated guideline for reporting systematic reviews. BMJ 2021;372:n71. doi: 10.1136/bmj.n71.
This work is licensed under CC BY 4.0. To view a copy of this license, visit https://creativecommons.org/licenses/by/4.0/

PROCESS 2025 Guideline Checklist			
**Topic**	**Item**	**Item description**	**Page Number**
**Title**	1	• The phrase 'case series' is included<br>• The focus of the research study is mentioned (e.g. patient population, setting, diagnosis, intervention, outcome etc.)	Y
**Key Words**	2	• Include three to six keywords that identify what is covered in the case series (e.g. patient population, setting, diagnosis, intervention, outcome etc.)<br>• Include 'case series' as one of the keywords<br>• Include the surgical subspecialty the case series pertains to as a keyword	Y
**Abstract**	3a	Introduction – briefly describe:<br>• Background<br>• Scientific rationale for this study<br>• Overarching theme of the case series<br>• Aims and objectives	Y

	3b	Methods – briefly describe:<br><br>● Sample size<br>● Timeframe of research<br>● Characteristics of study design (e.g. prospective/retrospective, single-/multi-centre, informal/formal, consecutive/non-consecutive, exposure-/outcome-based sampling, clinical/population-based etc.)<br>● Inclusion and exclusion criteria	Y
	3c	Results – briefly describe:<br><br>● Outcomes of the intervention/management strategy<br>● Analysis - narrative or statistical (report any statistical testing, although mostly inappropriate in case series studies)	Y
	3d	Conclusion – briefly describe:<br><br>● Key findings and take-home messages<br>● Impact on future clinical practice<br>● Direction of future research	Y
	3e	Present a structured abstract<br><br>● Informal case series – introduction, case presentations (brief description of each case) and discussion/conclusion<br>● Formal case series – introduction, methods, results and discussion/conclusion	Y<br>Formal case series
**Highlights**	4	● Convey the key findings of the research study in 3 to 5 bullet points	•Reconstruction of heel soft-

			tissue defects remains a surgical challenge due to limited local options.<br><br>• This study combines a systematic review and a retrospective single-centre case series.<br><br>• Modified rhomboid flaps achieved complete healing in all four patients without complications.<br><br>• Local flaps provide durable

			coverage and preserve sensation for small heel defects.
**Artificial Intelligence (AI)**<br><br>(some journals may prefer this in the methods and/or acknowledgments section and it should also be declared in the cover letter)	5	Declaration of whether any AI was used in the research and manuscript development<br><br>• If no, proceed to item 6.<br>• If yes, proceed to item 5a	No. Artificial intelligenc e (ChatGPT, OpenAI, GPT-5, 2025 version) was used for language editing and grammar correction of the manuscript .
	5a	**Purpose and Scope of AI Use**<br><br>• Precisely state why AI was employed (e.g. development of research questions, language drafting, statistical analysis/summarisation, image annotation, etc).<br>• Was generative AI utilised and if so, how?	

		• Clarify the stage(s) of the reporting workflow affected (planning, writing, revisions, figure creation).<br>Confirmation that the author(s) take responsibility for the integrity of the content affected/generated.	
	5b	**AI Tool(s) and Configuration**<br><br>• Name each system (vendor, model, major version/date).<br>• State the date it was used<br>• Specify relevant parameters (e.g. prompt length, plug-ins, fine-tuning, temperature).<br>• Declare whether the tool operated locally on-premises, or via a cloud API and any integrations with other systems.	
	5c	**Data Inputs and Safeguards**<br><br>• Describe categories of data provided to the AI (patient text, de-identified images, literature abstracts).<br>• Confirm that all inputs were de-identified and compliant with GDPR/HIPAA.<br>• Note any institutional approvals or data-sharing agreements obtained.	
	5d	**Human Oversight and Verification**<br><br>• Identify the supervising author(s) who reviewed every AI output.<br>• Detail the process for fact-checking, clinical accuracy checks	

		• State whether any AI-generated text/figures were edited or discarded.<br>• Acknowledge the limitations of AI and its use	
	5e	**Bias, Ethics and Regulatory Compliance**<br><br>• Outline steps taken to detect and mitigate algorithmic bias (e.g. cross-checking against under-represented populations).<br>• Affirm adherence to relevant ethical frameworks.<br>Disclose any conflicts of interest or financial ties to AI vendors.	
	5f	**Reproducibility and Transparency**<br><br>• Provide the exact prompts or code snippets (as supplementary material if lengthy).<br>• Supply version-controlled logs or model cards where possible.<br>• If applicable, state repository, hyperlink or digital object identifier (DOI) where AI-generated artefacts can be accessed, enabling attempts at independent replication of the query/input.	

**Introduction**	6	**Introduction**<br>comprehensively describe:<br><br>● Relevant background and scientific rationale for case series with reference to key scientific literature<br>● Overarching theme (e.g. common patient population, setting, diagnosis, intervention, outcome etc.)<br>● Aims and objectives<br>● At the end of the introduction, refer to the PROCESS 2025 publication by stating: 'This case series has been reported in line with the PROCESS guidelines [include citation]'	Y 2.3
**Methods**	7a	**Participants**<br>comprehensively describe:<br><br>● Relevant participant characteristics (e.g. demographics, comorbidities, ASA score, severity of surgery, urgency of surgery, smoking status, tumour staging etc.) and if relevant, exposure(s) of the participants (e.g. COVID-19)<br>● Subsequent inclusion and exclusion criteria with clear definitions<br>● Approach to selecting patients (e.g. consecutive/non-consecutive, exposure-/outcome-based, formal/informal etc.)<br>Methods used to ensure de-identification of patient information	Y Table 1

	7b	**Recruitment**<br>comprehensively describe:<br><br>● Sources of recruitment (e.g. physician referral, electronic health record etc.) Any monetary incentivisation of patients for recruitment and retention should be declared; clarify the nature of any incentives provided	Y 2.3<br>No incentives or compensation were provided for inclusion in this study.
	7c	**Pre-intervention patient optimisation:**<br><br>● Lifestyle (e.g. weight loss, nutritional support, exercise, smoking cessation etc.)<br>● Medication review (e.g. anticoagulation, oral hypoglycemics, insulin, oral contraceptive pill etc.)<br>● Pre-surgical stabilisation/preparation (e.g. treating hypothermia/-volemia/-tension, ICU care, nil by mouth, bowel preparation etc.) Other (e.g. psychological support, pre-operative education/counselling etc.)	Partial Surgical debridement and infection control are described, but optimisation measures are not explicitly detailed.
	7d	**Interventions**<br>comprehensively describe:<br><br>● Type of intervention (e.g. pharmacological, surgical, physiotherapy, psychological etc.)<br>● Aim of intervention (preventative/therapeutic)<br>● Concurrent treatments (e.g. antibiotics, analgesia, antiemetics, venous thromboembolism prophylaxis etc.)	Y 3.1

	7e	**Intervention specifics**<br>comprehensively describe:<br><br>● Rationale for the treatment offered<br>● Techniques involved in the administration of the intervention<br>● Time to intervention<br>● For pharmacological therapies, include details such as formulation, dosage, strength, route and duration<br>● For surgical intervention, include details on anaesthesia, patient positioning, preparation used, equipment needed, devices, sutures, surgical stage etc.<br>● Degree of novelty of surgical technique/device (e.g. 'first in human' or 'first in this context')<br>● Manufacturer and model of any medical devices used	Y 2.3
	7f	**Operator details**<br>comprehensively describe:<br><br>● Relevant training, specialisation and operator's experience (e.g. average number of the relevant procedures performed annually, independent, needs direct/indirect supervision etc.)<br>● Learning curve for technique<br>● Requirement for additional training<br>● Collaboration with other specialities (e.g. hybrid cardiac surgery)	Y 2.3

	7g	**Quality control**<br>comprehensively describe:<br><br>● Measures taken to reduce inter- or intra-operator/operation variation, ensure quality and maintain consistency between cases (e.g. independent observers, lymph node counts, standard surgical technique etc.)<br>● Any specific disparities between cases	Y 2.3, end
	7h	**Post-operative care and follow-up**<br>comprehensively describe:<br><br>● Post-operative care (e.g. patient education, post-operative medications, early mobilisation, targeted physiotherapy, early enteral nutrition, early removal of catheters/drains, psychological therapy etc.)<br>● Follow-up timeframes (e.g. first follow-up post-discharge, follow-up duration at the time of submission etc.) and frequency<br>● Follow-up setting (e.g. home via phone/video consultation, primary care, secondary care etc.)<br>● Follow-up method (e.g. history, clinical examination, blood tests, imaging etc.)<br>● Follow-up personnel (e.g. operating surgeon)<br>● Any specific long-term surveillance requirements (e.g. imaging surveillance of endovascular aneurysm repair, clinical/ultrasound examination	Y 2.3 & 3.1

		of regional lymph nodes for skin cancer etc.)<br>● State if any participants were lost to follow-up and why	
	7i	**Analysis**<br><br>● Narrative or statistical (report any statistical testing, although mostly inappropriate in case series studies)	Narrative synthesis.
**Results**	8a	**Participants**<br>comprehensively describe:<br><br>● Number of patients involved<br>● Patient characteristics (e.g. demographics, comorbidities, ASA score, severity of surgery, urgency of surgery, smoking status, tumour staging etc.) and if relevant, exposure(s) of the participants (e.g. COVID-19)	Y, Table 1

		● Include table showing baseline patient characteristics	
	8b	**Deviation from the initial management plan** comprehensively describe:<br><br>● Any changes to the planned intervention with rationale<br>● If appropriate, include a suitable schematic diagram	No deviation from the planned surgical management occurred.
	8c	**Outcomes and follow-up** comprehensively describe:<br><br>● Expected versus attained clinician assessed outcome, providing reference to scientific literature used to inform expected outcomes (e.g. core outcome set)<br>● If appropriate, include patient-reported outcomes (e.g. quality-of-life)<br>● Use of validated outcome measures<br>● Time of outcome occurrence<br>● Percentage of patients lost to follow-up with rationale	Partial. Clinical outcomes and follow-up duration are reported, but no measures were used.

	8d	**Intervention adherence and compliance** comprehensively describe:<br><br>● Assessment of patient's adherence and tolerability of intervention and post-operative instructions (e.g. avoiding heavy lifting/strenuous activity, tolerance of chemotherapy/pharmacological agents etc.)<br>● Impact on long-term applicability of intervention in clinical practice	Compliance is not explicitly described in the manuscript. But all patients complied with postoperative instructions regarding weight-bearing restriction and dressing care.

	8e	**Complications and adverse events** comprehensively describe:<br><br>● Precautionary measures taken to prevent complications (e.g. antibiotic/venous thromboembolism prophylaxis)<br>● Complications and adverse events (e.g. blood loss, wound infection, deep vein thrombosis, pulmonary embolism etc.), categorised in accordance with the Clavien-Dindo classification<br>● Timing of adverse events<br>● Mitigation for adverse events (e.g. blood transfusion, wound care, re-exploration/revision surgery etc.)<br>● If relevant, whether complications or adverse events were discussed locally (e.g. morbidity and mortality meetings)<br>● If appropriate, whether complications or adverse events were reported to the relevant national agency or pharmaceutical company<br>● Specify time to discharge following completion of intervention and whether this was within the expected timeframe or not (if not, why not)<br>● Where applicable, specify the 30-day post-operative and long-term morbidity/mortality<br>● State if there were no complications or adverse events	Partial.<br><br>Complications are described narratively, but no formal Clavien–Dindo classification was applied.

**Discussion**	9a	**Key results**<br>comprehensively describe:<br>● Key results<br>● Include table showing key results	Y<br>Discussion paragraph 1 & Table 1
	9b	**Scientific context and implications**<br>comprehensively describe:<br>● Relevant literature and if appropriate, similar published studies<br>● Implications for clinical practice and guidelines (e.g. NICE)<br>● Comparison to current gold standard of care<br>● Relevant hypothesis generation	Y<br>paragraph 1 & 2
	9c	**Strengths**<br>comprehensively describe:<br>● Strengths of the study<br>● Any multidisciplinary or cross-speciality relevance	Y<br>paragraph 2

	9d	**Weaknesses and limitations** comprehensively describe:<br>● Weaknesses and limitations of the study, with potential impact on results and their interpretation<br>● Deviations from protocol, with reasons<br>● For novel techniques or devices, outline any contraindications/alternatives and potential risks/complications if applied to a larger population	Y paragraph 3
	9e	**Directions for future research** comprehensively describe:<br>● Impact on future research and clinical practice<br>● Questions that have arisen as a result of the study<br>● Alternative study design(s) best suited to address these questions	Y conclusions, end
	9f	**Cost** comprehensively describe:<br>● Economic implication(s)<br>● Justify cost if intervention more expensive than current gold standard of care<br>● Any cheaper alternatives	No cost analysis was performed, as the procedure uses standard surgical materials and procedures.

**Conclusions**	10a	**Key conclusions**<br><br>● Outline the key conclusions from this study	Y Conclus ions
	10b	**Rationale**<br><br>● Explain the rationale behind those conclusions	Y
	10c	**Future work**<br>briefly describe:<br><br>● Any questions arisen from the study<br>● Any differences in approach to patient diagnosis or management which authors might adopt in future similar studies	Y
**Patient and/or Carer Perspective**	11	● Where appropriate, the patient(s)/carers(s) should be given the opportunity to share their perspective on the intervention(s) they received (e.g. sharing quotes from a consented, anonymised interview or questionnaire)	Partial. 3.1

**Informed Consent**	12	• The authors must provide evidence of consent, where applicable, and if requested by the journal<br>• State the method of consent at the end of the article (e.g. verbal or written)<br>• If not provided by the patients, explain why (e.g. death of patient and consent provided by next of kin). If the patients or family members were untraceable then document the tracing efforts undertaken.	Y
**Additional Information**	13a	• State any conflicts of interest	Y
	13b	• State any sources of funding (e.g. grant details)<br>• Role of funder	Y
	13c	Other relevant disclosures<br>• State any author contributions and acknowledgments<br>• If appropriate, give details of institutional review board and ethical committee approval<br>• Disclose whether the case has been presented at a conference or regional meeting	Y

**Clinical Images and Videos**	14	• Where relevant and available, include clinical images to help demonstrate the cases pre-, peri- and post-intervention (e.g. radiological, histopathological, patient photographs, intraoperative images etc.)<br>• Where relevant and available, include a link (e.g. Google Drive, YouTube etc.) to the narrated operative video to highlight specific techniques or operative findings<br>• Ensure all media files are appropriately captioned and indicate points of interest to allow for easy interpretation	Y Figures 3 & 4
**Referencing the Checklist**	15	• Include reference to the PROCESS 2023 publication by stating: 'This case series has been reported in line with the PROCESS Guideline' at the end of the methods section and include citation in the references section	Y
